# The hands’ default location guides tactile spatial selectivity

**DOI:** 10.1073/pnas.2209680120

**Published:** 2023-04-04

**Authors:** Stephanie Badde, Tobias Heed

**Affiliations:** ^a^Department of Psychology, Tufts University, Medford, MA 02155; ^b^Cognitive Psychology, Department of Psychology, University of Salzburg, 5020 Salzburg, Austria; ^c^Centre of Cognitive Neuroscience, University of Salzburg, 5020 Salzburg, Austria

**Keywords:** tactile, spatial selectivity, body posture, tactile motion, temporal perception

## Abstract

Our skin is a two-dimensional sheet that can be folded into a multitude of configurations due to the mobility of our body parts. Parts of the human tactile system might account for this flexibility by being tuned to locations in the world rather than on the skin. Using adaptation, we scrutinized the spatial selectivity of two tactile perceptual mechanisms for which the visual equivalents have been reported to be selective in world coordinates: tactile motion and the duration of tactile events. Participants’ hand position—uncrossed or crossed—as well as the stimulated hand varied independently across adaptation and test phases. This design distinguished among somatotopic selectivity for locations on the skin and spatiotopic selectivity for locations in the environment, but also tested spatial selectivity that fits neither of these classical reference frames and is based on the default position of the hands. For both features, adaptation consistently affected subsequent tactile perception at the adapted hand, reflecting skin-bound spatial selectivity. Yet, tactile motion and temporal adaptation also transferred across hands but only if the hands were crossed during the adaptation phase, that is, when one hand was placed at the other hand’s typical location. Thus, selectivity for locations in the world was based on default rather than online sensory information about the location of the hands. These results challenge the prevalent dichotomy of somatotopic and spatiotopic selectivity and suggest that prior information about the hands’ default position —right hand at the right side—is embedded deep in the tactile sensory system.

## Significance

When we touch objects in the dark, we must take body posture into account to determine the objects’ location. This could be achieved by mechanisms that are selective for tactile stimuli located at specific locations in the world irrespective of their location on the skin. Using adaptation, an established behavioral paradigm to probe the properties of neural mechanisms, we indeed found evidence for spatial selectivity beyond skin locations. Yet, this selectivity reflected the hands’ typical not their actual location in space. Our result challenges the widely held idea that sensory events are coded exclusively in topographic reference frames and suggests that prior information about the location of the hands is embedded in the tactile sensory system.

Famously, tactile neurons in primary somatosensory cortex are somatotopically tuned: they selectively respond to tactile stimulation of circumscribed skin regions (1). However, due to the mobility of our body parts, there is no direct mapping between locations on the skin and locations in the world. One way in which such a mapping could be implemented at an early stage of the perceptual process are neurons that selectively respond to tactile stimulation at specific world locations such as in the observer’s right hemispace. Indeed, several high-level visual features are encoded by neural mechanisms selective in world coordinates (2–4). Yet, it remains unknown whether or not spatial selectivity in the tactile system depends on the position of the stimulated body part (5), which is a prerequisite of selectivity for locations in the world. This gap holds at the level of single neurons and at the level of neural mechanisms that underlie the perception of tactile features.

There are three possible outcomes when probing the spatial selectivity of a neural mechanism underlying the perception of a tactile feature. First, it is possible that somatotopic spatial selectivity prevails throughout the tactile system and across tactile features. For example, a neuronal population that encodes tactile motion direction might do so exclusively for one specific skin site such as the right hand, independent of the hand’s position. Second, a neural mechanism could be spatiotopically tuned, that is, selective for locations in the world. For example, a neuronal population that encodes tactile motion direction might do so exclusively for tactile stimulation elicited by touching an object located to the right of the observer, independent of the body part that touches the object. Spatiotopic selectivity in the tactile system implies that spatial selectivity depends on online sensory posture information because the location of a touched object needs to be deducted from the position of the body part touching the object. Yet third, there is another possibility to how spatial selectivity might bridge between locations on the skin and the external world. Tactile perception is strongly affected by the default position of the touched limbs (6, 7). For example, perceptual errors are much more frequent when the hands are crossed (8, 9), i.e., when the hands are not at their default locations in space. Thus, spatial selectivity for locations in the world might be guided by prior rather than online sensory information about the location of the limbs (10).

This study scrutinized the spatial selectivity of the neural mechanisms that underlie the perception of directional tactile motion and the duration of tactile events. We chose these two features because the mechanisms underlying their visual counterparts are reportedly selective in world coordinates (2, 11). To test spatial selectivity, we used adaptation aftereffects, that is, changes in perception following prolonged exposure to a stimulus. Adaptation is a well-established behavioral paradigm to probe the properties of neural mechanisms. The adapted mechanism is considered spatially selective if adaptation aftereffects emerge only for test stimuli presented at specific locations. The relationship between the adapted locations and those showing aftereffects indicates the reference frame of spatial selectivity. For example, aftereffects limited to the adapted skin site indicate somatotopic spatial selectivity. Some previous tactile adaptation studies tested the spatial selectivity of the mechanisms underlying the perception of tactile numerosity, orientation, and duration (12–15). Yet, these studies did not test for the possibility that default rather than online sensory information about the position of the limbs underlies spatial selectivity beyond locations on the skin. Adaptation has also been used to uncover the tuning of several spatial tactile features. For example, tactile distances are encoded in skin-based coordinates (16), but tactile apparent motion is encoded by mechanisms tuned to motion directions in world coordinates (17). Yet, the reference frame in which a feature is encoded does not necessarily correspond to the spatial selectivity of the feature-encoding mechanism. For example, a population of neurons might respond to tactile motion from right to left in world coordinates but only when this motion is sensed on the right hand—implying spatiotopic tuning for motion direction combined with somatotopic spatial selectivity.

**Fig. 1.**
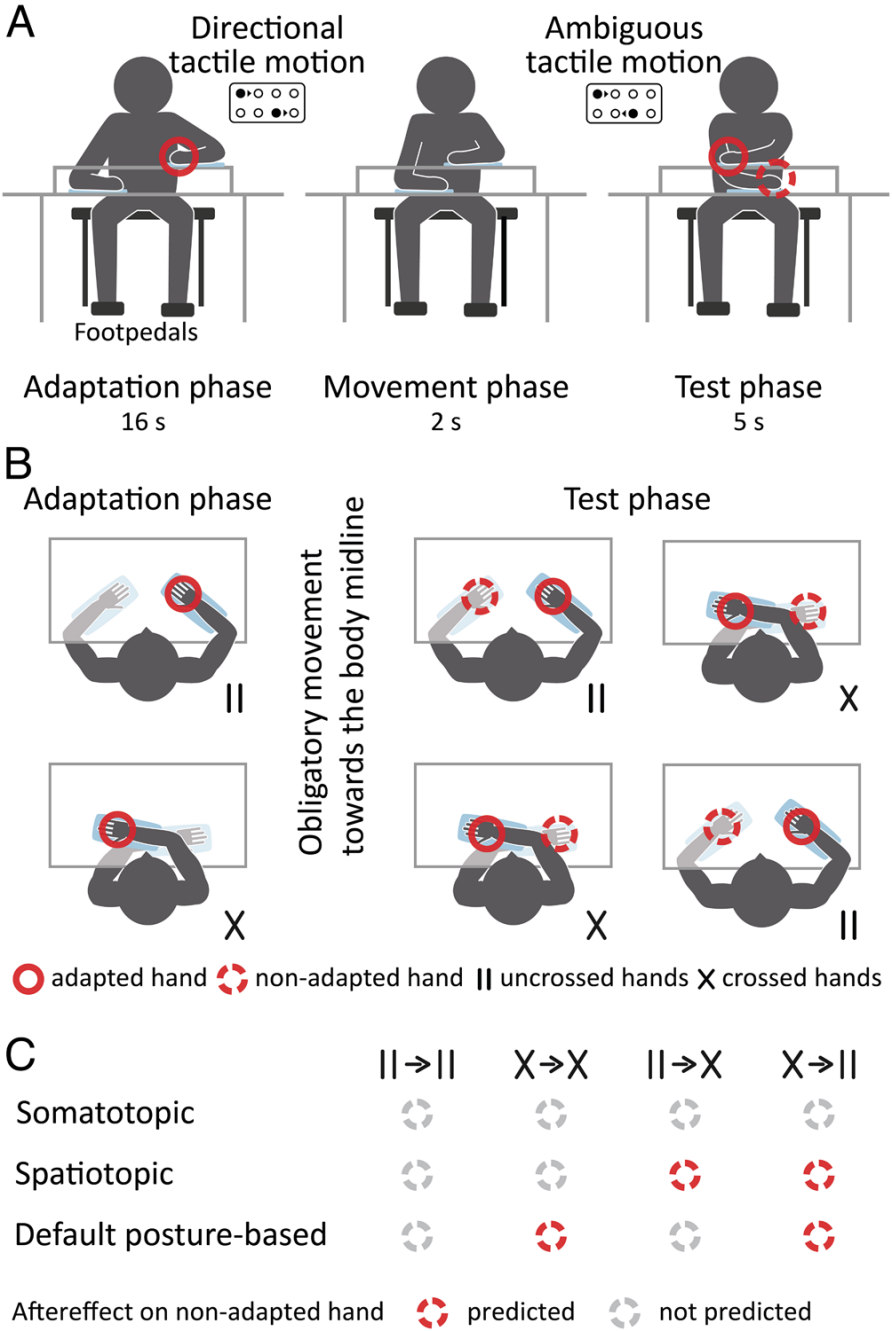
Procedure, design, and hypotheses. (*A*) Experimental procedure. Participants sat at a table and positioned one arm on the table surface and the other arm on a second surface 20 cm above. One finger of each hand (Experiment 1) or both wrists (Experiment 2) rested on tactile stimulators embedded in cushions with a slippery underside. Each stimulator held eight individually controllable pins; successively raising subsets of pins elicited either directional or ambiguous apparent tactile motion percepts (*Insets*). In Experiment 1, participants were adapted to directional motion on one hand. During the test phase, the participants used foot pedals to continuously report the perceived direction of ambiguous motion stimuli either on the adapted or the other hand. In Experiment 2, the participants were adapted either to ambiguous tactile motion or to a static tactile stimulus. In the test phase, the participants indicated which of two short ambiguous motion stimuli—one on either the adapted or the other hand, the other one on the abdomen—they perceived as longer. After each adaptation phase, the participants aligned their hands with the body midline before they moved their hands into position for the test phase (movement phase). (*B*) Experimental design. During adaptation and test phases, the hands were positioned either uncrossed (II) or crossed (X), resulting in four different sequences (II→II, X→X, II→X, and X→II). The test stimulus was either applied to the adapted or the nonadapted hand. (*C*) Predictions for adaptation aftereffects on the nonadapted hand depending on the underlying type of spatial selectivity and hand position during adaptation and test phases. Somatotopic spatial selectivity predicts no aftereffects on the nonadapted hand; spatiotopic selectivity predicts aftereffects on the nonadapted hand whenever it is placed in the same world location as the adaptor, i.e., when the hands change position between adaptation and test phases but not when hand position remains constant; default posture-based spatial selectivity predicts aftereffects on the nonadapted hand whenever it was located at the adapted hand's default location during the adaptation, i.e., only when the hands were crossed during the adaptation.

Our experimental design disambiguates somatotopic, spatiotopic, and default posture-based spatial selectivity of the adapted neural mechanisms: The stimulated hand as well as participants’ hand position—uncrossed or crossed—varied independently across adaptation and test phases (Fig. 1 *A* and *B*). If a mechanism is purely somatotopically tuned, i.e., spatially selective for locations on the skin, adaptation should affect the perception of test stimuli on the adapted hand but not of test stimuli on the nonadapted hand (Fig. 1*C*). In turn, if a mechanism is spatiotopically tuned, i.e., spatially selective for locations in the world, adaptation should affect the perception of test stimuli on any hand placed at the location the adapted hand occupied during the adaptation phase. Thus, spatiotopic tuning, which means that online sensory information about the position of the hands guides spatial selectivity, predicts aftereffects on the nonadapted hand whenever the hands swap positions between adaptation and test phases (Fig. 1*C*). Finally, if spatial selectivity of the tested mechanisms does account for body posture but does so in terms of the default rather than actual position of the hands, adaptation should affect perception at any hand placed at the adapted hand’s default location during adaptation. That is, if spatial selectivity for locations beyond the skin is guided by the hands’ default position, aftereffects on the nonadapted should emerge whenever the hands were crossed during adaptation—regardless of the hands being uncrossed or crossed during the test phase (Fig. 1*C*).

## Results

**Fig. 2.**
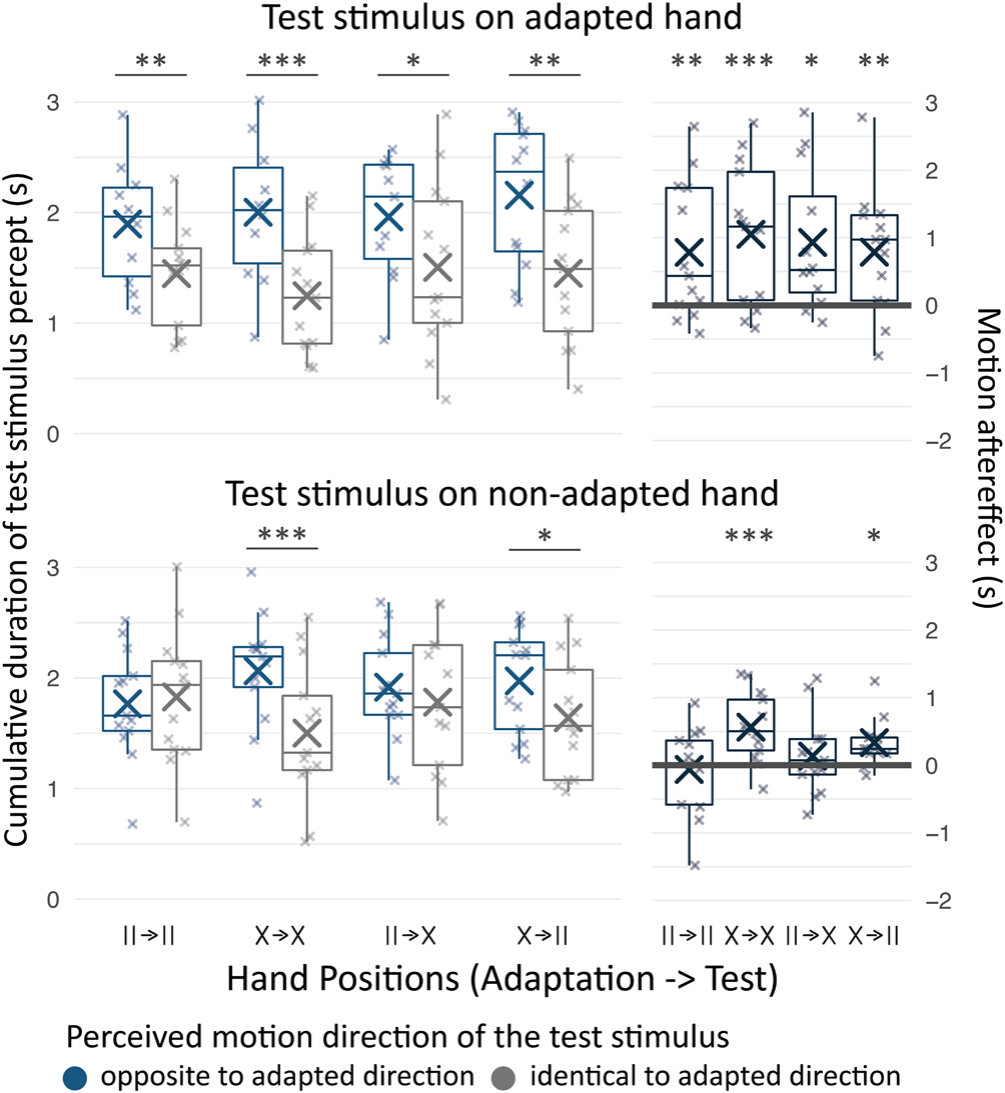
Motion direction adaptation aftereffects. Cumulative time for which the 5-s long ambiguous test stimulus was perceived as either moving in the same (gray) or opposite (blue) direction as the adaptor (*Left panels*) and individual motion aftereffects, i.e., differences between the cumulative durations of both motion percepts (opposite—identical; *Right* panels). Boxplots show the distribution of participant-level means (center line, median; box limits, upper and lower quartiles; whiskers, minimum and maximum limited to 1.5× interquartile range beyond the quartiles); large markers show group means, small markers show participant-level mean values. Data are split by the location of the test stimulus (adapted or nonadapted hand) and the sequence of hand positions during adaptation and test phases. Significance stars (*: α=0.05 , **: α=0.01 , ***: α=0.001 ) are identical across *Left* and *Right* panels and indicate the results of contrast analyses testing aftereffects in each condition against zero.

In Experiment 1, participants were adapted to directional tactile apparent motion. When the adaptation is successful, participants typically perceive subsequently presented direction-ambiguous test stimuli as moving in the opposite direction as the adapting stimulus (18, 19) (Fig. 1*A*; see figure caption and Methods for experimental details). Accordingly, we measured tactile motion aftereffects as the amount of time for which a 5 seconds-long, direction-ambiguous test stimulus was perceived as moving in the opposite rather than in the same direction as the adapting stimulus. Reliable motion aftereffects emerged for test stimuli on the adapted hand [χ^2^(1) = 17.8, *P* < 0.001] statistically independent of hand positions during adaptation and test phases [χ^2^(5) = 2.15, *P* = 0.542; Fig. 2, *Upper* panels]. Critically, significant aftereffects also emerged on the nonadapted hand. This transfer was evident only if the hands had been crossed during adaptation [χ^2^(5) = 13.5, *P* = 0.004; Fig. 2, *Lower* panels], whether the hands were uncrossed (z = 14.91, *P* = 0.020; Fig. 2, *Lower* panels X→II) or crossed (z = 3.78, *P* < 0.001; Fig. 2, *Lower* panels X→X) during the test phase. In contrast, no significant aftereffects emerged on the nonadapted hand if the hands had been uncrossed during the adaptation phase (uncrossed test phase, II→II: z = −0.47, *P* = 0.639, crossed test phase, II→X: z = 0.96, *P* = 0.337; Fig. 2, *Lower* panel). In sum, motion aftereffects reliably occurred at the adapted hand, yet the presence of aftereffects on the nonadapted hand excludes strictly somatotopic tuning of the adapted mechanism. Moreover, transfer of motion adaptation from one hand to the other was not contingent on matching world locations of adaptor and test stimulus, which is the case when the hands swap their positions between adaptation and test phase. This renders spatiotopic selectivity of the adapted motion-encoding mechanism unlikely (Fig. 1*C*). Rather, aftereffects transferred from one hand to the other only if the hands were crossed during the adaptation phase. That is aftereffects were present on the nonadapted hand only if it rested at the adapted hand’s default location during the adaptation. Together, the results suggest that directional tactile motion is encoded by mechanisms that are somatotopically tuned and take the hands’ default position into account.

**Fig. 3.**
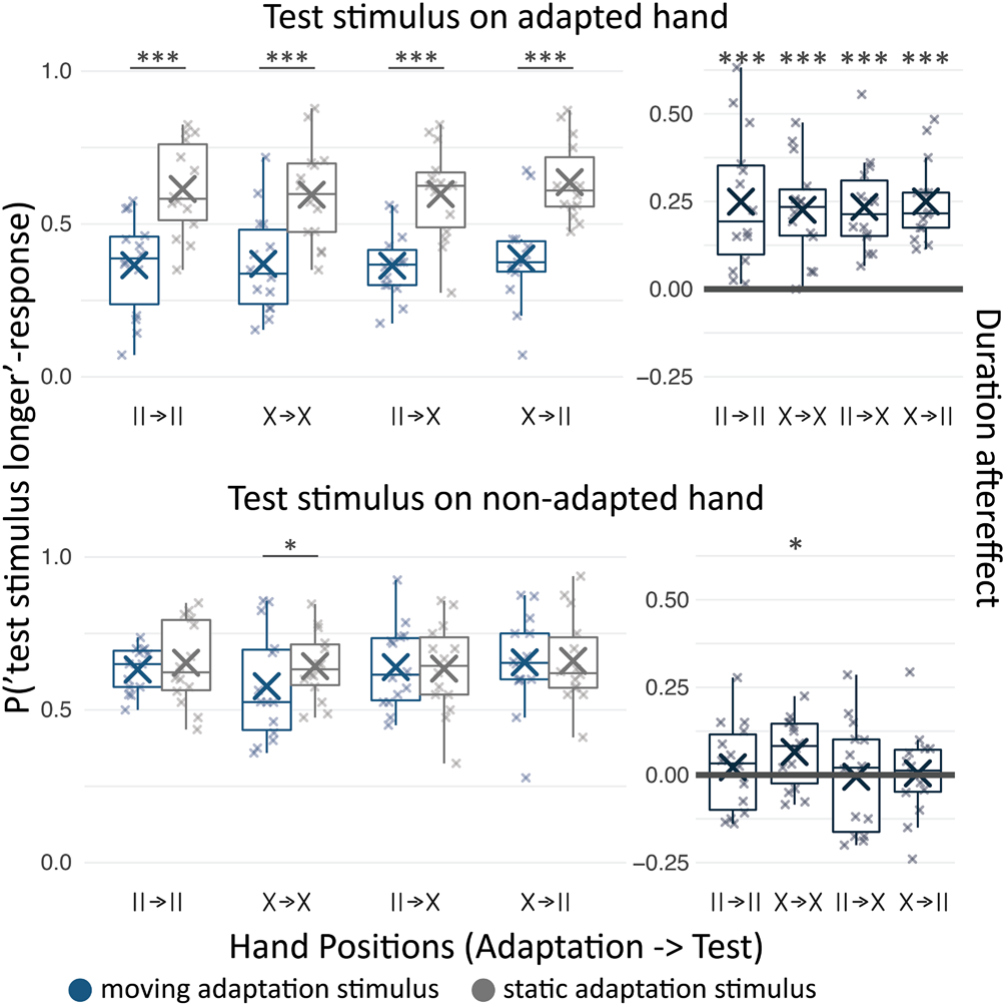
Tactile event duration adaptation aftereffects. Probability of perceiving a test stimulus presented on one of the wrists as longer than a standard stimulus presented on the abdomen (*Left panels*) following adaptation to direction-ambiguous tactile motion (blue) and adaptation to a static tactile stimulus (gray) as well as duration aftereffects, i.e., the difference between the two adaptation conditions (moving—static, *Right* panels). All other conventions are as in Fig. 2.

In Experiment 2, we asked whether this spatial selectivity is specific to tactile motion or extends to other tactile features. Particularly, we were interested in temporal perception because in the visual domain the neural mechanisms underlying the timing of events are reportedly spatially selective in a spatiotopic reference frame (2). Thus, we tested for duration adaptation aftereffects (13, 20) on the adapted and nonadapted hand using the same design as in Experiment 1. We adapted participants to direction-ambiguous tactile motion; participants typically perceive subsequently presented tactile stimuli as shorter than they would without adaptation (12, 13, 20). The perceived duration of tactile stimuli on the hands was measured as the probability of perceiving a test stimulus on one of the hands as longer than a tactile standard stimulus on the abdomen. To assess adaptation aftereffects, we compared duration percepts following adaptation to direction-ambiguous tactile motion with duration percepts following adaptation to a static tactile stimulus. The comparison between the two adaptation conditions controlled for potential differences in perceived duration between tactile stimuli on the hands and the abdomen. Again, significant aftereffects emerged for the adapted hand [χ^2^(1) = 232.85, *P* < 0.001], statistically independent of hand position [χ^2^(5) = 0.36, *P* = 0.949; Fig. 1 *D*, *Left* panels], as well as for the nonadapted hand if the arms had been crossed during adaptation and test phases (X→X, z = 2.07, *P* = 0.038; Fig. 1 *D*, *Right* panels). Hence, Experiment 2 speaks against purely somatotopic selectivity of the mechanism underlying the timing of tactile events given that adaptation affected perception on the nonadapted hand. The results also speak against spatiotopic spatial selectivity because there is no evidence for transfer of adaptation across hands when they swapped locations between adaptation and test phase; thus, aftereffects were not tied to locations in the world (Fig. 1*C*). Evidence for default posture-based spatial selectivity was mixed for the tested temporal mechanism because duration judgments for the nonadapted hand were significantly affected only in one of the two conditions that involved crossed hands during the adaptation phase. Notably, participants gave a single, binary response in Experiment 2, whereas they reported their motion direction percepts over several seconds in Experiment 1, leading to a higher amount of measurement noise in Experiment 2. When we increased the sensitivity of our analysis by including only those trials in which the test stimulus was longer than the standard stimulus (see *SI Appendix* for the rationale behind this analysis), aftereffects on the nonadapted hand were present whenever the hands were crossed during the adaptation period (*SI Appendix*, Fig. S3). That is, there is some support for spatial selectivity based on the default position of the hands, but none for spatial selectivity based on the hands' actual position. Thus, the spatial tuning of the mechanisms that time tactile events does not fit the dichotomy of somatotopic and spatiotopic reference frames Fig. 3.

## Discussion

Our study reveals that tactile spatial selectivity of the mechanisms underlying the perception of directional tactile motion relies on default rather than online sensory posture information in addition to somatotopic spatial tuning. If the hands were placed in their default position—right hand at the right side—adaptation exclusively affected tactile motion perception at the adapted skin location. Yet, motion adaptation led to perceptual aftereffects on the nonadapted hand if the non-adapted hand rested at the default location of the adapted hand during adaptation. This result is inconsistent with strictly somatotopic spatial selectivity because adaptation transferred across skin locations. It is also inconsistent with spatiotopic spatial selectivity because transfer did not depend on the world location of the tactile stimuli. Instead, our findings suggest the existence of motion-sensitive neural mechanisms that encode both touch to a hand and touch located in this hand’s default hemispace. This connection between a hand and its hemispace implies that prior information about the typical position of the hands is embedded deep in the tactile system. A similar result emerged for the mechanisms underlying the perception of tactile event duration. Here, too, results were incompatible with both strictly somatotopic and spatiotopic spatial selectivity, but mostly compatible with default posture-based selectivity. This correspondence between the spatial tuning of the mechanisms underlying these two features, both of which have been associated with spatiotopic spatial selectivity in vision (2, 11), indicates a spatial organization beyond somatotopic and spatiotopic reference frames in the tactile system.

At first, the spatial tuning we report here may seem paradoxical. The adapted perceptual mechanisms are spatially selective in a reference frame that conflicts with the typical assumption of a topographical organization that is ubiquitous in tactile, visual, attentional, and motor research. Nonetheless, a mechanism that selectively encodes touch on a hand and touch located in the hand’s default hemispace can explain multiple well-known perceptual phenomena, because this paired selectivity can explicate how a touch on a crossed hand can lead to perceptual, cognitive, and motor effects typically associated with a tactile stimulus on the other hand. Tactile stimuli to crossed hands i) are sometimes perceived at hands or feet positioned in the default hemispace of the stimulated limb (7), ii) initially bias visual attention to the default rather than correct location of the stimulated hand (21), iii) lead to trajectories curved toward the touched limb’s default hemispace when they are the target of saccades (22, 23) or pointing movements (24), iv) interfere with directional apparent motion percepts (25, 26), and v) are difficult to order spatiotemporally (8, 9, 27–29). Prevalent theories posit that such influences of posture on perception and action reflect errors, or intermediate steps, in the calculation of spatiotopic coordinates (6, 8, 21, 27, 30–32). In this view, the corresponding representations are transient and associated with rare perceptual and motor errors. In contrast with this view, the present study provides no evidence for a relation between hand position effects and accurate spatiotopic representations of touch based on online sensory information. Instead, our findings reveal that the tactile system natively incorporates the hands’ default location. Thus, the “activation” of the right hemispace by a touch on the (crossed) right hand that initially seemed like a fleeting, intermediate step (8, 21, 31, 32) is embedded deep in the tactile system.

One possible reason for incorporating prior rather than online sensory information about the current position of the hands is that sensory information is not always available. Effects like the rubber hand illusion (33) demonstrate that vision strongly affects where we perceive our hands to be located. Yet, humans tend to look at the environment rather than at their own bodies, which renders visual information about the location of the hands unavailable. Proprioceptive information about hand position is much noisier and decays in the absence of active movements (34). Thus, the use of prior information about hand position could serve as a fallback—one that is reliable in most everyday situations as humans seldom use a hand located in the opposite hemispace.

We can only speculate whether the link between a hand and its default location is inherent to the neural organization of the tactile perceptual system or formed through experience. On the one hand, limb crossing is rather unusual across our evolutionary heritage. On the other hand, tactile perceptual errors provide evidence that knowledge about the hands’ default location might be acquired or strengthened through early visual experience. Congenitally blind individuals identify tactile stimuli on crossed or rotated hands with the same accuracy as stimuli on uncrossed and unrotated hands (35–37). In contrast, individuals who lost their sight later in life show the same tendency to confuse the spatiotemporal order of tactile stimuli to crossed hands as sighted participants (35). Thus, both our genetic make-up and experience may contribute to the strong connection between a hand and its typical location in space.

There are two ways in which adaptation aftereffects on the nonadapted hand could arise in our experiments: One possibility is that tactile adaptation also affected the hand that would usually be located at the world location of the adaptor. The other possibility is that tactile adaptation of a crossed hand affected tactile perception with the other hand because that hand was located at the adapted hand’s default location. Our design does not allow us to decide between these two alternatives, but previous results favor the latter mechanism: Tactile stimuli on a crossed hand initially facilitate visual discrimination at the touched hand’s default location (21) and are sometimes erroneously attributed to the hand or foot placed in that hand’s default hemispace (7). Both findings imply that the adapted hand’s default spatial location is relevant. In any case, either of the two scenarios implies that it is not online sensory but prior information about the location of the hands that determines spatial selectivity beyond locations on the skin.

This study scrutinized the spatial selectivity of the neural mechanisms underlying perception of two tactile features and thus targeted their spatial representation presumably at early processing stages. An accurate spatiotopic representation of these tactile features could still be constructed via other mechanisms than spatial selectivity. Moreover, we cannot exclude the possibility that spatial selectivity for world locations exists at later stages in the tactile processing hierarchy that cannot be reached by adaptation or for features other than the ones tested here. However, it should be noted that there is currently little evidence for the automatic emergence of a spatiotopic representation of touch based on online sensory information about the current posture of the body. Hand position effects in tactile perception, which traditionally have been interpreted as evidence for an accurate spatiotopic representation of touch (6, 30), can also be explained by spatial tuning based on the default position of the hands (7). Additionally, neuroimaging studies (38) and neuronal recordings in tactile-visual integration areas (39, 40) have provide evidence that some parietal regions align visual with tactile receptive fields, rather than being spatiotopically tuned for touch. It is thinkable that localizing a touched object in space is a two-step process that does not involve a spatiotopically organized spatial representation of tactile features. In a first step, the identity of the touched limb could be identified, potentially based on both the somatotopic and default posture-based spatial tuning revealed here. Then, kinesthetic and visual information might be used to identify the world location of the stimulated body part without linking this information back to the touch. This idea is consistent with the surprising finding that participants confabulate world locations for tactile stimuli when they assign a tactile stimulus to the wrong, moving hand (41). Moreover, this account fits with the suggestion that tactile spatial processing proceeds independent of posture information based on how humans perceive trajectories drawn along the skin (42, 43). In summary, the spatial selectivity revealed here might mean that the brain overcomes the lack of a consistent mapping between the skin and space (44) through an approximation of a spatiotopic representation that relies on prior information about where our body parts normally reside.

## Materials and Methods

### Participants.

Fourteen participants (all right-handed; six males; aged 19 to 37 y, average 25 y) completed Experiment 1. Two additional participants aborted the experiment early and data of one additional participant were excluded because they disclosed to have consumed amphetamines prior to one session. Another fourteen participants (all right-handed; two males; aged 19 to 39 y, average 26 y) completed Experiment 2. Three additional participants aborted the experiment early. Sample sizes were determined based on the literature, specifically based on studies reporting tactile adaptation effects (16, 18) and studies using adaptation to investigate spatial selectivity in vision (2, 3). All participants reported normal or corrected-to-normal vision and absence of tactile and motor impairments. Participants were naïve to the purpose of the study. They received course credit or were compensated with 7 Euro/h. The study was approved by the ethics board of the German psychological society and all experiments were conducted in accordance with the general guidelines laid down in the Declaration of Helsinki (7th revision, 2013, except preregistration). Participants gave a written informed consent prior to the beginning of the experiment.

### Apparatus and Stimuli.

Participants sat at a table and rested one arm on the table surface and the other arm on a second surface raised by 20 cm (Fig. 1*A*). They positioned their hands palm down; the lower arms oriented approximately parallel to the torso. Flat cushions with a slippery underside were attached to the ventral side of each forearm. Homolog fingertips of each hand (Experiment 1 and 2) or both wrists (10 of 14 participants in Experiment 2) rested on tactile stimulators (QuaeroSys Medical Devices, Stuttgart, Germany) that were embedded in the cushions. Over the course of the experiment, the site of stimulation was varied between index, middle, and ring finger, to counteract numbing due to prolonged tactile stimulation. In Experiment 2, numbing set in earlier than in Experiment 1, probably due to a higher stimulus speed. Therefore, we changed the stimulation site to the ventral side of the wrist after testing that the size of the adaptation effect did not critically depend on the adapted skin site. In Experiment 2, an additional stimulator was attached to participants’ abdomen, fixed centrally below the belly button with a foam cushion held by a wide elastic ribbon. We chose the abdomen because tactile stimulation at other locations along the body midline such as the forehead or the sternum leads to bone conduction which renders tactile stimuli audible.

Each stimulator held eight, individually controllable pins with a diameter of 0.8 mm that were arranged in a 4 × 2 array with an interpin distance of 2.5 mm (Fig. 1 *A*, *Insets*). Activated pins protruded by 2.5 mm and retracted again with a frequency of 100 Hz. Disjunct sets of two pins, one in each column of the pin array, were successively activated to elicit apparent motion directed toward the body (i.e., lateral motion across the finger pad moving toward the thumb), directed away from the body (i.e., lateral motion across the finger pad moving toward the pinky), or moving with ambiguous direction. A directional motion percept was elicited by shifting the locations of the two raised pins (black circles in Fig. 1*A*) in the same direction with every update of the set of raised pins; an ambiguous motion percept was elicited by shifting the locations of the two pins in opposite directions. Apparent movement speed was 62.5 mm/s in Experiment 1, and 130 mm/s in Experiment 2, realized by updating the set of raised pins every 40 and 20 ms, respectively. Even though the stimulation iterated through a sequence of four sets of two raised pins, the percept resembled constant motion rather than tactile sweeps because of the spatial separation between the two raised pins. For static stimuli (Experiment 2), the pins protruded by 4 mm at the beginning of the stimulation and remained in this position until the end of stimulation.

Participants wore earplugs and headphones that emitted white noise to mask any auditory cues elicited by the tactile stimulators. Participants’ feet rested on custom-made foot pedals, and they responded by lifting either the forefoot or the heel. They were instructed to fixate a central marker positioned on the farther horizontal edge of the upper surface so that participants saw the position of the upper arm while fixating a steady target in all conditions. Experiments were controlled by Presentation, version 16.1 (Neurobehavioral Systems), which interfaced with custom-built hardware to drive stimulators and record responses.

### Task and Procedure.

#### Experiment 1—Motion adaptation.

Each trial began with the presentation of directional tactile motion on one finger for 16 s (adaptation phase; Fig. 1*A*). During this period, participants indicated their tactile motion percept using the foot pedals. They could choose between motion toward the body (right heel), motion away from the body (right forefoot), and ambiguous motion (left forefoot or heel; participants could choose a response mapping at the beginning of the experiment, almost all decided on the one just outlined). The participants gave a foot pedal response whenever their motion percept changed and reconfirmed consistent motion percepts at self-paced, regular intervals. We chose this response mode because piloting had shown that continuously lifting their toes or heel was too strenuous.

Once the adaptation stimulus ended, the participants aligned their hands with the body midline and then moved their arms into position for the test phase (movement phase, Fig. 1*A*). These arm movements had to be completed within 2 s and were executed in every trial, whether hand position during the test phase was identical to or different from hand position during the adaptation phase. Visual markers on the upper surface indicated the target locations for the hand movements, but participants hardly relied on these markers for their movements. They practiced the movements extensively at the beginning of the experiment and practiced again whenever the posture sequence changed. The experimenter supervised the training and visually controlled whether participants consistently arrived at the target positions during the experiment. To support timely and correct arm movements, the experimenter stood in front of the participant and simultaneously executed the same arm movements throughout the experiment.

During the subsequent test phase, an ambiguous motion stimulus was presented for 5 s either on the adapted finger or on the homologous finger of the other hand. The participants again indicated their motion percept using the foot pedals. The next trial started 4 s after the end of the test stimulus to minimize carry-over effects.

The direction of the tactile motion presented during the adaptation phase, the adapted hand, and the tested hand varied pseudo-randomly across trials. The four possible sequences of hand positions during adaptation and test phases (uncrossed then uncrossed, II→II, uncrossed then crossed, II→X, crossed then uncrossed, X→II, and crossed then crossed, X→X) were manipulated block wise; block order was counterbalanced across participants. The stimulated finger (index, middle, ring) varied randomly from block to block. Overall, the participants completed 40 trials for each of the eight critical experimental conditions (2 tested hands × 4 hand position sequences), resulting in a total of 320 trials, divided into 20 blocks of 16 trials each. The participants split the experiment into three sessions of about 1.5 to 2 h each.

#### Experiment 2—Duration adaptation.

During the adaptation phase, either a direction-ambiguously moving or a static tactile stimulus was presented. In this experiment, a click sound indicated the end of the adaptation and the beginning of the movement phase because otherwise participants might have missed the end of the static adaptation stimulus.

During the test phase, two direction-ambiguous tactile motion stimuli were successively presented, one on the abdomen and one on either the adapted or the nonadapted hand. The two stimuli were presented in random order with a 1-s long interstimulus interval. Stimulation on the abdomen, the standard stimulus, lasted 800 ms; stimulation on a hand, the test stimulus, lasted either 640 or 960 ms, based on piloting data (S1). Participants used the foot pedals to indicate which of the two stimuli they had perceived as longer, the first (right forefoot) or the second (right heel) stimulus (participants had the option to choose the response assignment, all of them preferred this assignment).

Experiment 2 comprised 640 trials divided into 80 blocks; the participants completed 40 trials for each of the 16 critical experimental conditions (2 adaptation types × 2 tested hands × 4 hand position sequences). Overall, the experiment took about 7.5 h, split into multiple sessions. The participants chose the number of blocks per session themselves. Most participants completed the experiment in five sessions of 1.5 h; two participants with stimulation on the fingers split the experiment into more sessions to avoid their fingers being numb at the end of the session. Blocks with static adaptation stimuli were always administered before blocks with moving adaptation stimuli to avoid carry-over effects from moving to static stimulus conditions.

All other experimental parameters were identical to those of Experiment 1.

### Data Analysis.

#### Experiment 1—Motion adaptation.

Responses that occurred later than 1.5 s after the end of stimulation were excluded from the analysis (0.3% of responses). We coded participants’ motion direction responses in the test phase relative to the direction of the motion stimulus in the adaptation phase. Thus, recoded responses indicate whether the ambiguous test motion was perceived as moving in the same or opposite direction as the adapting stimulus, or in an ambiguous direction. We constructed timelines sampled at 100 Hz from the intermittently given responses, effectively accounting for potential changes of the motion percept in time steps of 10 ms. From these timelines, we extracted the cumulative duration of each motion percept during the test phase. Responses indicating ambiguous motion percepts were rare (mean duration of ambiguous motion percepts: 109 ± 23 ms), and we therefore did not include them in the analysis. Durations were submitted to a linear mixed-effects regression model with random intercepts and three fixed effects: perceived motion direction (levels: identical vs. opposite to the adapted direction), tested hand (levels: test stimulus presented on adapted vs. nonadapted hand), and hand position sequence (levels: II→II, II→X, X→II, and X→X). Adaptation aftereffects correspond to the difference between the cumulative durations of test motion percepts moving in the same and the oppositive direction as the adapting stimulus.

#### Experiment 2—Duration adaptation.

Participants’ binary responses were recoded as 1 if the response indicated that the participant perceived the test stimulus on a hand as longer than the standard stimulus on the abdomen and 0 otherwise. Responses were submitted to a generalized linear mixed regression model with binomial distribution family and logit link function. We included random intercepts and three fixed effects: adaptation type (levels: ambiguous motion vs. static stimulus), tested hand (levels: adapted vs. nonadapted hand), and hand position sequence (levels: II→II, II→X, X→II, and X→X). Adaptation effects correspond to the difference in the proportion of trials in which the test stimulus at the hand was perceived as longer than that at the abdomen between trials following a moving adaptation stimulus and trials following a static adaptation stimulus.

For both experiments, contrast analyses were used to test for adaptation aftereffects in individual conditions. We report two-sided *P*-values that were corrected for multiple comparisons using Benjamini and Hochberg’s procedure.

All statistical analyses were conducted using R, version 4.1.2 (45).

## Supplementary Material

Appendix 01 (PDF)

## Data Availability

Anonymized (.txt files) data have been deposited in osf (https://osf.io/pv2xf/) (46).
