# Supplementary material for: The hands’ default location guides tactile spatial selectivity
Source: Proc Natl Acad Sci U S A. 2023 Apr 4;120(15):e2209680120. doi: 10.1073/pnas.2209680120 (PMC10104573; doi:10.1073/pnas.2209680120)
Supplement: Supplementary file 1 — Appendix 01 (PDF) [file pnas.2209680120.sapp.pdf]

PNAS

Supporting Information for
The hands' default location guides tactile spatial selectivity.

Stephanie Badde & Tobias Heed

Corresponding author: Stephanie Badde
Email: stephanie.badde@tufts.edu

This PDF file includes:

Supporting text
Figures S1 to S3

Supporting Information Text

Duration Adaptation Aftereffects – Pilot

To check whether we could replicate the aftereffects of adaptation to an ambiguously moving tactile stimulus on the perceived duration of tactile stimuli in our design, we conducted a small pilot study. We additionally used this study to select a subset of test durations as the number of conditions in main experiment did not permit mapping out a full psychometric function. The hands remained in an uncrossed position throughout the experiment and test stimuli were always applied to the adapted, right hand. Five test stimulus durations, 720, 960, 880, 640, and 800 ms, were presented 28 times in each adaptation condition. All other experimental parameters were identical to Expt. 2.

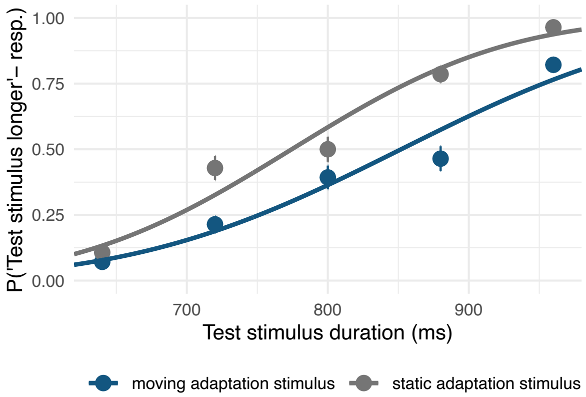

Fig. S1. Results pilot experiment on tactile event duration aftereffects. The proportion of trials in which the test stimulus on the right hand was perceived as longer than the standard stimulus on the abdomen is shown as a function of test stimulus duration separately for trials following a moving adaptation stimulus (blue) and a static adaptation stimulus (grey). Cumulative Gaussian distribution functions were fitted separately to data from each condition (lines).

Duration Adaptation Aftereffects – Simulation

Adaptation leads to a perceived shortening of the duration of tactile events. Thus, adaptation aftereffects are smaller for shorter durations than for longer ones, whether the aftereffect is constant or proportional to the stimulus duration (see Fig. S2 for a simulation and Fig. S1 for pilot data). The intuition behind this general effect is as follows: adaptation leads to a decrease in the perceived duration of the test stimuli. This will result in a measurable effect only if without the adaptation the test stimulus would have been perceived as longer than the standard stimulus but after the adaptation it is perceived as shorter. If the test stimulus would have been perceived as shorter without the adaptation, the effect of the adaptation will be present but cannot be measured with the two-alternatives-forced choice task used in our study. This latter case is more likely to occur for shorter than for longer test stimuli. The presence of a measurable effect is most likely for test stimuli slightly longer than the standard stimulus as for very long stimuli a

perceived shortening of the test stimulus might not be sufficient to perceive it as shorter than the standard stimulus.

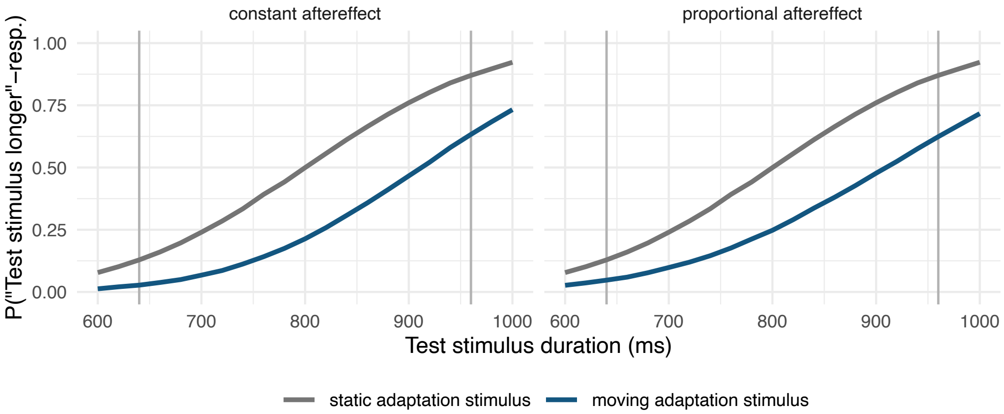

Fig. S2. Simulation of constant and proportional duration aftereffects. Simulated proportion of trials in which the test stimulus was perceived as longer than the standard stimulus at the abdomen as a function of test stimulus duration. Responses simulated without adaptation aftereffects (corresponding to a static adaptation stimulus in our design; grey curves) and responses simulated assuming either a constant adaptation aftereffect (blue curve, left panel) or an aftereffect proportional to the test stimulus duration (blue curve, right panel) are shown. Vertical grey lines indicate the test stimulus durations used in Expt. 2. R code for this simulation is provided on OSF.

Duration Adaptation Aftereffects – Exploratory Analysis

The above outlined relation between the size of the adaptation aftereffect and the duration of the test stimulus implies that test stimuli with a longer duration provide a more sensitive test of adaptation aftereffects. Thus, we conducted an analysis of only those trials in which the test stimulus was longer than the standard stimulus in Expt. 2.

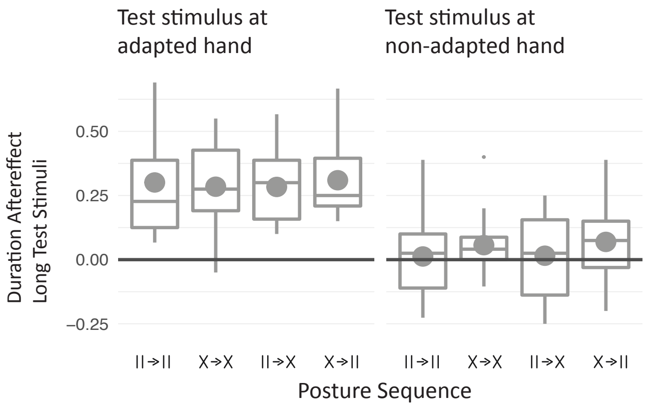

Fig. S3. Duration Aftereffects for Long Test Stimuli. Duration aftereffects, the difference in the probability of perceiving a test stimulus presented on one of the wrists as longer

than a standard stimulus presented on the abdomen (Expt. 2) between adaptation to moving and static stimuli restricted to trials in which the test stimulus was longer than the standard stimulus. Boxplots show the distribution of participant-level means (center line, median; box limits, upper and lower quartiles; whiskers, minimum and maximum limited to 1.5x interquartile range beyond the quartiles); circular markers show group means. Data are split by the location of the test stimulus (adapted or non-adapted hand) and the sequence of hand positions during adaptation and test phases. Positive values indicate duration adaptation aftereffects.
